# Ocular Dirofilariasis, a Case Report

**Published:** 2010-09

**Authors:** MR Fallah Tafti, A Hajilary, H Siatiri, MB Rokni, I Mobedi, Gh Mowlavi

**Affiliations:** 1 Eye Research Center, Tehran University of Medical Sciences, Tehran Iran; 2 Department of Medical Parasitology & Mycology, School of Public Health, Tehran University of Medical Sciences, Iran

**Keywords:** Dirofilariasis, Ocular, Human, Case Report, Iran

## Abstract

Accidental infection with animal filarial worms in humans is a dilemma for clinicians and parasitologists throughout the world. To date a variety of such rare parasitoses have been reported mostly in tropics and subtropics. Human dirofilariasis is among those unusual zoonotic infections that occasionally have been observed in the eye and in subcutaneous areas exhibiting with nodule formation. Filarial worms are transmitted to humans through invertebrate biological vectors such as certain species of mosquitoes. The present report describes a peculiar case of ocular dirofilariasis in a 49-year-old man resident in Iran.

## Introduction

The genus *Dirofilaria* belongs to the family Onchocercidae and subfamily Dirofilariinae of the order Spirurida (1). It can infect dogs and other mammals, including cats, foxes, sea lions, wolves, and otters (2). *Dirofilaria* sp. known as a mosquito transmitted filarial nematode of domestic and wild carnivores occasionally may infect humans as an accidental zoonotic infection. Insects ingest microfilariae from the definitive host and the larvae become mature through two molts within the insect. The L3 stage is the infective filariform responsible to develop an infection in vertebrate hosts through the mosquito blood meal (3). Based on the absence or presence of external longitudinal cuticular ridges, two subgenera of *D. dirofilaria* and *D. nuchtiella* are well-recognized (4). *D. nochtiella repens*, which sometimes occurs in the conjunctiva of humans in the old world and D*. tenuis* with similar status in the southern United States formerly was mentioned as *D. conjunctiva* (5). From the perspective of ophthalmology however, documented dirofilariasis has been observed in the eyelid, periorbital region, subconjunctival tissues (6) and rarely in intravitreal region (7).

Initially, the presence of so-called dog heartworm, *D. immitis* in the right heart of a dog was stated by Francesco Birago in River Valley of Italy in 1626^8^. However, the first report of human occurrence was in gastrosplenic ligament of a Hungarian woman in 1879 (9). Ever since, in parallel with quick development in common knowledge, fast global growing of communication and greater awareness in the medical community, the number of *Dirofila* infections have been increased globally (10). There are also several documented reports of ophthalmic infections worldwide including America, Europe, and Australia (11). Eosinophilic meningitis in human has also attributed to these filarial nematodes as etiological agent diagnosed by immunofluorescence and arthus hypersensitivity (12). Inclusively several clinical cases of human dirofilariasis, including 270 pulmonary cases have been reported up to the end of the second millennium (13).

## Case report

A 49-year-old Iranian man, resident in Tehran, Iran of a good socio-economic level was primarily referred to an ophthalmologist with redness, tearing, blepharospasm, swelling of lids, photophobia, and cystic swelling on the temporal side of bulbar conjunctiva of right eye. betamethasone eye drop was prescribed 4 times daily as the case was diagnosed allergic conjunctivitis. Three days later, patient referred to Farabi Eye Hospital; with severe manifestations. Ocular examination showed palpebral swelling with hyperemia and swelling directly over the lateral rectus muscle insertion with Congestion of episcleral vessels. The cornea at temporal side was slightly opaque, anterior chamber, lens vitreous and retina was unremarkable.

Visual acuity and IOP was normal. Diagnosis and treatment of episcleritis was done.

**Fig. 1:**
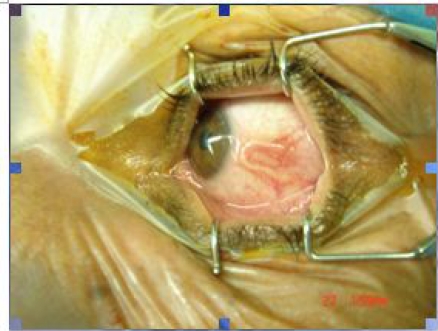
*Dirofilaria repens* lying within sclera forming U shape granuloma (Source: Authors)

One-month later patient came back to the hospital exhibiting a hard U shape granulated tissue at the lesion site (Fig. 1), without any remarkable disorder in his eye. The lesion was explored under local anesthesia and after incision of granuloma a 1.5 cm. White roundworm was extracted from area and was sent to parasitology department for diagnosis.

### Specimen identification

**Fig. 2:**
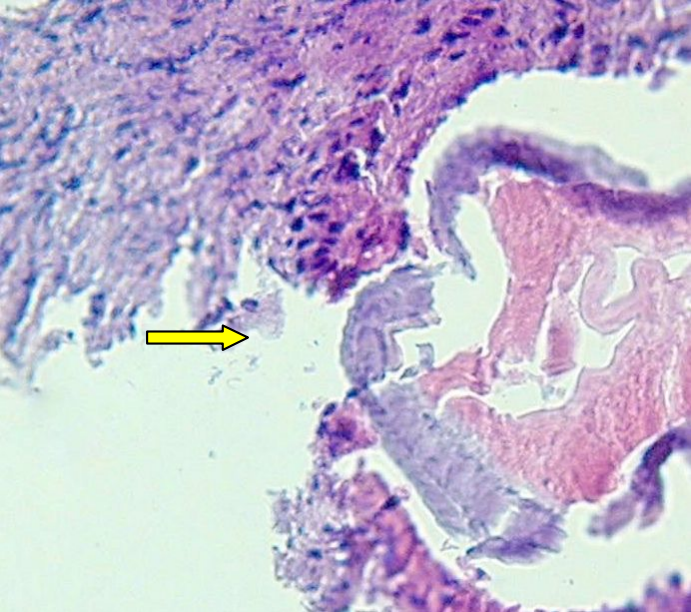
Cross section showing longitudinal ridges indicating *Dirofilaria repens*(40X) (Source: Authors)

Extracted worm was recognized as immature *D. repens* of 18 mm length and 280-µm width. Identification was conducted using authentic diagnostic clues (4) based upon the morphological appearance completed with observing longitudinal cuticular ridges in cross sections. Due to unwilling extra manipulation and staining processes, the best typical taxonomic feature was not attained successfully as it could be obviously illustrated. However, it was not so hard to browse for pieces of longitudinal cuticular ridges among the slide sections to confirm differentiation of D*. repens* from *D. immitis* confidently (Fig. 2)*.*

## Discussion

To date, 12 confirmed cases of human dirofilariasis have been reported in Iran, scattered across 11 provinces. Three (a case of testicular hydrocele and two pulmonary cases) have been attributed to *D. immitis* and the rest (four subcutaneous, two ocular and three subconjunctival cases) to *D. repens* (14, 15). There have also been two possible cases: the individual, with an ocular problem possibly caused by *D. conjunctivae* (16), and a patient with a swollen right forearm, possibly caused by *D. Repens* (17). Meanwhile dirofilariasis in carnivores have been documented so far in Iran (18). In a recent publication, prevalence of this zoonotic filarial worm among those red foxes and jackals with proximity to human residing areas has posed the importance of this issue (19). Because of the free roaming behavior of definitive hosts, the main source of infection looks available for mosquitoes such as Culicidae members.

In contrast with many parasitic infections in humans, dirofilariasis seems hardly in relation with personal hygienic conditions when the biology of Culicidae vectors is considered in many parts of the world. This is also can be claimed that the rate of human dirofilariasis might be more than the present rate in the literature. Notable occurrences of ocular dirofilariasis within the entire records in Iran will drag the mind toward a great need of comprehensives investigation on the parasite eco biological traits itself, and the factors contributing in human transmission.

## References

[1] Anderson RC (2000). Nematode parasites of vertebrates, their development and transmission.

[2] Beaver PC (1965). Orihel TC.Human infection with filariae of animals in the United States. Am J Trop Med Hyg..

[3] Muller R (2002). Worms and human diseases.

[4] Wayne M, Meyers RonaldC (2000). Neafie, Aileen, Marty, Douglas J. Wear. Pathology of Infectious Diseases. Helminthiasis.

[5] Gutierrez Y (1990). Diagnostic Pathology of Parasitic Infections with Clinical Correlations. Philadelphia.

[6] Font R L, Neafie R C, Perry HD (1980). Subcutaneous Dirofilariasis of the Eyelid and Ocular Adnexa: Report of six cases. Archives of Ophthalmology..

[7] Gorezis S, Psilla M, Asproudis I, Peschos D, Papadopoulou C, Stefaniotou M (2006). Intravitreal dirofilariasis: a rare ocular infection. Orbit.

[8] Roncalli Amici R (2001). The history of Italian parasitology. Vet Parasitol..

[9] Bruijning CF (1981). Human dirofilariasis: A report of the first case of ocular dirofilariasis in the Netherlands and a review of the literature. Trop Geogr Med..

[10] Ciferri F (1982). Human pulmonary dirofilariasis in the United States: a critical review. Am J Trop Med Hyg.

[11] Gavin J Stringfellow MB BS, Ian C Francis FRANZCO, Minas T Coroneo Franzco, John Walker (2002). Orbital dirofilariasis. Clin Experimen Ophthalmo.

[12] Dobson C, Welch J S (1974). Dirofilariasis as a cause of eosinophilic meningitis in man diagnosed by immunofluorescence and Arhus hypersensitivity. Trans R Soc Trop Med Hyg..

[13] Vieira C, Montoya M, Agudelo S, Velez D, Simon F (2008). Human antibody response to a 56-kDa purified excretory/secretory product of Dirofilaria immitis. Trop Med International Health..

[14] Rokni MB (2008). The present status of human helminthic diseases in Iran. Ann Trop Med Parasitol..

[15] Siavashi MR (1995). Human cutaneous dirofilariasis in Iran. Iranian J Med Sci..

[16] Rohani S, Athari A (2003). Ocular dirofilariasis in Iran: a case report. Medical Journal of Islamic Republic of Iran..

[17] Negahban S, Daneshbod Y, Atefi S, Daneshbod K, Sadjjadi SM, Hosseini SV (2007). *Dirofilaria repens* diagnosed by the presence of microfilariae in fine needle aspirates: a case report. Acta Cytol..

[18] Sadighian A (1969). Helminth parasites of stray dogs and jackals in Shahsavar area, Caspian region, Iran. J Parasitol..

[19] Meshgi B, Eslami A, Bahonar AR, Kharrazian-Moghadam M, Gerami-Sadeghian A (2009). Prevalence of parasitic infections in the red fox (Vulpes vulpes) and golden Jackal. IJVR..

